# “*They Just Tell Me to Abstain:”* Variable Access to and Uptake of Sexual and Reproductive Health Services Among Adolescents Living With HIV in Kenya

**DOI:** 10.3389/frph.2021.644832

**Published:** 2021-04-26

**Authors:** Sarah Lawrence, Hellen Moraa, Kate Wilson, Immaculate Mutisya, Jillian Neary, John Kinuthia, Janet Itindi, Edward Nyaboe, Odylia Muhenje, Tai-Ho Chen, Benson Singa, Christine J. McGrath, Evelyn Ngugi, Pamela Kohler, Alison C. Roxby, Abraham Katana, Lucy Ng'ang'a, Grace C. John-Stewart, Kristin Beima-Sofie

**Affiliations:** ^1^Department of Global Health, University of Washington, Seattle, WA, United States; ^2^Department of Paediatrics, University of Nairobi, Nairobi, Kenya; ^3^Division of Global HIV & Tuberculosis, U.S. Centers for Disease Control and Prevention, Nairobi, Kenya; ^4^Department of Research and Programs, Kenyatta National Hospital, Nairobi, Kenya; ^5^Centre for Clinical Research, Kenya Medical Research Institute, Nairobi, Kenya; ^6^Department of Child, Family, and Population Health Nursing, University of Washington, Seattle, WA, United States; ^7^Department of Medicine, University of Washington, Seattle, WA, United States; ^8^Department of Epidemiology, University of Washington, Seattle, WA, United States; ^9^Department of Pediatrics, University of Washington, Seattle, WA, United States

**Keywords:** adolescent HIV, adolescent sexual and reproductive health, mixed methods, adolescents, implementation science

## Abstract

**Background:** To improve holistic care for adolescents living with HIV (ALHIV), including integration of sexual and reproductive health services (SRHS), the Kenya Ministry of Health implemented an adolescent package of care (APOC). To inform optimized SRH service delivery, we sought to understand the experiences with SRHS for ALHIV, their primary caregivers, and health care workers (HCWs) following APOC implementation.

**Methods:** We completed a mixed methods evaluation to characterize SRHS provided and personal experiences with access and uptake using surveys conducted with facility managers from 102 randomly selected large HIV treatment facilities throughout Kenya. Among a subset of 4 APOC-trained facilities in a high burden county, we conducted in-depth interviews (IDIs) with 40 ALHIV and 40 caregivers of ALHIV, and 4 focus group discussions (FGDs) with HCWs. Qualitative data was analyzed using thematic analysis. Facility survey data was analyzed using descriptive statistics.

**Results:** Of 102 surveyed facilities, only 56% reported training in APOC and 12% reported receiving additional adolescent-related SRHS training outside of APOC. Frequency of condom provision to ALHIV varied, with 65% of facilities providing condoms daily and 11% never providing condoms to ALHIV. Family planning (FP) was provided to ALHIV daily in 60% of facilities, whereas 14% of facilities reported not providing any FP services to ALHIV. Screening and treatment for STIs for adolescents were provided at all clinics, with 67% providing STI services daily. Three key themes emerged characterizing experiences with adolescent SRHS access and uptake: (1) HCWs were the preferred source for SRH information, (2) greater adolescent autonomy was a facilitator of SRH discussions with HCWs, and (3) ALHIV had variable access to and limited uptake of SRHS within APOC-trained health facilities. The primary SRHS reported available to ALHIV were abstinence and condom use education. There was variable access to FP, condoms, pregnancy and STI testing, and partner services. Adolescents reported limited utilization of SRHS beyond education.

**Conclusions:** Our results indicate a gap in SRHS offered within APOC trained facilities and highlight the importance of adolescent autonomy when providing SRHS and further HCW training to improve SRHS integration within HIV care for ALHIV.

## Introduction

There are 2.1 million adolescents living with HIV (ALHIV) globally, the majority in sub-Saharan Africa (SSA). An estimated 6% percent of the world's population of ALHIV live in Kenya (1). Adolescents (ages 10–19 years) face a number of challenges as they transition from childhood to adulthood, including adjusting to physical and psychosocial transformations, as well as increasing independence (2). For ALHIV, these challenges likely intensify preexisting stressors related to HIV infection, such as HIV status disclosure and increasing personal responsibility for treatment adherence (3). The initiation of sexual relationships and associated challenges of living with a lifelong communicable disease that can be sexually transmitted further complicates this time for many ALHIV (4). Evidence suggests ALHIV have better health outcomes if adolescent friendly services (AFS) [i.e., services that are accessible, acceptable, equitable, appropriate and effective for adolescents (5, 6)] are provided. However, specialized programs have been slow to respond to these needs (3, 7). Evidence-based interventions and support are critical for improving ALHIV uptake of HIV care and sexual and reproductive health services (SRHS) during this critical time of change and development (8).

In Kenya, adolescents have limited sexual health knowledge (9), further increasing the risk of sexual transmission of HIV to new partners as well as unintended pregnancy. In 2018, more than half (54%) of the nation's sexually active adolescent females had an unmet need for family planning (10). A systematic review among ALHIV in SSA, including Kenya, found high rates of sexual risk-taking behaviors, including inconsistent condom use, transactional sex, and multiple partners during the last year (11). Findings from a recent study on modes of HIV transmission among youth in Kenya revealed the majority of new HIV infections among older adolescents are likely sexually acquired (12). These findings highlight the importance of improving SRHS for ALHIV.

SRHS and HIV care have often developed parallel infrastructures and constrained the ability to meet patient needs, but the benefits of integrating these services are widely documented (13–17). Integration of SRHS within HIV care can include offering both services at one site or ensuring health care workers (HCWs) have the knowledge and skills to provide information and referrals to locations where needed services are offered. Integration of these services provides an important opportunity to meet the health needs of ALHIV, however, it is not yet a common practice for ALHIV from SSA (18). HCWs in Kenya have reported barriers to providing SRHS to young people, including limited SRH knowledge themselves, staff shortages, inadequate training, and language differences (slang used by youth not understood) (19). Additionally, some HCWs in Kenya do not believe adolescents should have access to contraceptives or other SRHS (19).

In response to the need for improved adolescent services, the Kenya Ministry of Health (MOH), developed a comprehensive package of clinical and psychological services for adolescents known as the Adolescent Package of Care (APOC). Better understanding of SRHS provided at APOC-trained facilities can identify implementation gaps and inform areas for improvement in scaling up SRH education, access, and uptake for ALHIV. Therefore, the goal of this project was to improve delivery of evidence-based SRHS by characterizing current knowledge, attitudes and practices around provision of SRHS for ALHIV from the perspectives of ALHIV, primary caregivers of ALHIV, and HCWs.

## Methods

### Study Design and Population

This mixed methods evaluation was nested within the Public Health Adolescent Services Evaluation (PHASE). PHASE is a national evaluation of adolescent HIV services in Kenya undertaken as a collaborative effort between the Kenya MOH, U.S. Centers for Disease Control and Prevention (CDC), the Kenya Medical Research Institute (KEMRI), and the University of Washington (UW). This specific analysis used surveys, in-depth interviews (IDIs) and focus group discussions (FGDs) to determine availability, integration, experiences, and uptake of SRHS for ALHIV as part of national APOC roll-out.

**Figure 1:**
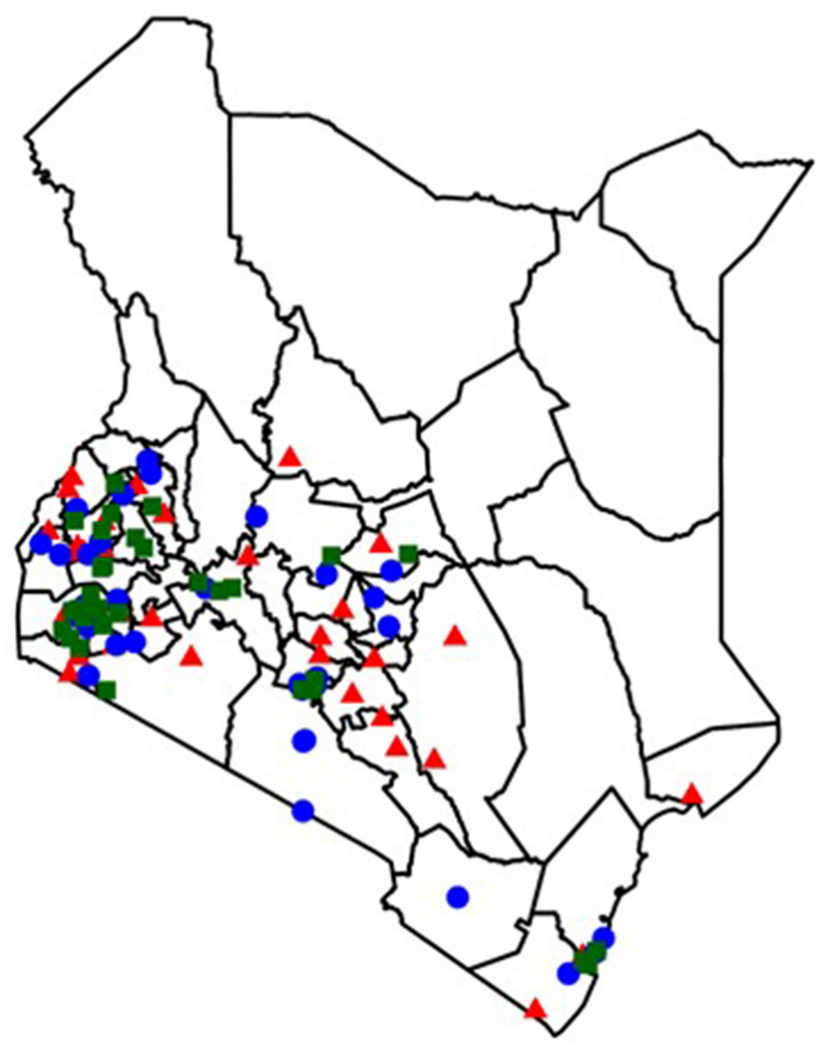
Distribution of facilities included in the facility survey. Facility distribution by estimated clinic size at the time of random selection. Blue Circle, small facilities (300–455 total HIV patients); Red Triangle, medium facilities (456–866 total HIV patients); Green Square, large facilities (≥ 867 total HIV patients).

We conducted surveys at 102 randomly selected HIV treatment facilities across Kenya selected from all large (>300 total patients in care) HIV facilities using electronic medical records (EMR) (Figure 1). Clinics were stratified into tertiles based on facility size, and an even number of small (300–455 total HIV patients), medium (456–866 total HIV patients) and large (≥867 total HIV patients) facilities were included. At a subset of 4 APOC-trained facilities in Homa Bay County, we also conducted IDIs with ALHIV and caregivers of ALHIV, and FGDs with HCWs. APOC includes a component on the provision of SRHS, including condoms, contraception, pregnancy testing, STI screening and management, and partner HIV testing. To capture variation in services by clinic size, facilities for qualitative data collection were purposively selected to include two medium and two large facilities. Small sites were excluded due to recruitment challenges with fewer adolescents receiving care.

Based on their knowledge of HIV services at the facility, facility managers either completed surveys, or referred surveys to HCWs with more direct experience caring for adolescents. HCWs who participated in FGDs were purposively sampled to include a broad range of HCW cadres to maximize the range of adolescent service provision experiences captured. Clinic staff contacted eligible ALHIV and their caregivers to initially explain the study. Interested individuals were referred to the study team for eligibility assessment and enrollment. Adolescents were eligible to participate if they were between 14 and 19 years of age, knew their HIV status, and were able to communicate in Dholuo, Kiswahili, or English. Caregivers were eligible if they were the primary caretaker of an ALHIV ages 10–17 and were able to communicate in Dholuo, Kiswahili, or English. Adolescents and caregivers were not required to participate as a dyad, although 16 dyads chose to. We targeted and enrolled 40 ALHIV and 40 caregivers.

### Data Collection

We collected data between February and May 2017. Study staff administered surveys to one HCW per selected facility. Surveys were administered by phone or in person, depending on HCW preference, and ascertained routine clinic practices related to the care of adolescents at their facility. Questions about SRHS included the frequency of provision of condoms (daily, weekly, biweekly, monthly, once every 3 months, or never), FP services, and screening and treatment for sexually transmitted infections (STIs) as well as adolescent-related SRHS training offerings within facilities. All IDIs and FGDs were conducted in health facilities using semi-structured discussion guides designed to elicit perspectives on five key topic areas that complemented survey data: (1) general adolescent HIV service provision and access, (2) HIV testing, (3) HIV disclosure, (4) HIV medication adherence, and (5) SRHS. HCW discussion guides also solicited views on personal experiences with APOC training and utilization of the APOC tools. Discussion guides were developed collaboratively by study team members based on literature reviews and expertise in HIV and SRH research. IDIs and FGDs were conducted by a trained interviewer in Dholuo, Kiswahili, English or a combination of these languages, depending on interviewee preference. IDIs ranged between 22 and 72 min and FGDs between 87 and 131 min in length. All IDIs and FGDs were audio recorded, transcribed verbatim and translated to English as needed. Targeted detailed summaries were written within 24–48 h of the IDI or FGD by the interviewer to capture the context and experience of each interview as well as summarize key topics.

### Data Analysis

The objective of this analysis was to characterize SRHS provided and current knowledge, attitudes, and practices around provision of SRHS for ALHIV from quantitative surveys and qualitative perspectives of ALHIV, primary caregivers of ALHIV, and HCWs. Facility surveys provided an overview of the landscape of services offered at facilities throughout Kenya. IDIs with ALHIV were analyzed to understand their experiences, motivations, and influences for accessing SRHS while IDIs with caregivers were analyzed to understand their attitudes toward adolescent SRHS and sexual health education and information. HCW FGDs were analyzed to understand HCW experiences providing SRHS to ALHIV and perceived influences and perspectives on SRHS for adolescents.

Facility survey data was analyzed using descriptive statistics in Stata version 14 (College Station, USA). IDIs and FGDs were analyzed using conventional content analysis (20) to produce a description of key concepts and themes arising within and between the individual categories represented in the interview guides. Transcripts were analyzed using a codebook that was developed iteratively by four members of the primary analysis team (HM, KBS, KW, SL) using both inductive and deductive approaches. Transcripts were imported into ATLAS.ti version 8 (Scientific Software Development GmbH, Berlin, Germany), which was used to manage data analysis. Transcripts were divided between team members and each transcript was coded independently by one member of the coding team using the final version of the codebook. Transcripts were exchanged and all previously coded transcripts were reviewed by another member of the team. Identified disagreements in code application were resolved through group discussion.

Ethical approvals were received from the KEMRI Scientific and Ethics Review Unit, and UW institutional review board. The project was also reviewed in accordance with the Centers for Disease Control and Prevention (CDC) human research protection procedures and was determined to be research, but CDC investigators did not interact with human subjects or have access to identifiable data for research purposes. All adult participants provided written informed consent while adolescents provided written assent with written parental permission to participate in the study. Participants were reimbursed for transportation costs and their time.

### Ethics Approval and Consent to Participate

The study was approved by the University of Washington Institutional Review Board (IRB) and the Kenya Medical and Research Institute Ethical Review Committee (ERC). All adult participants provided written informed consent while adolescents provided written assent with written parental permission to participate in the study.

## Results

### Facility Characteristics

Provision of adolescent SRHS, particularly the frequency of services offered, varied across the 102 health facilities (Table 1). Of surveyed facilities, 56% reported receiving training in APOC for HCWs at their facility, and 11% reported receiving additional SRHS training outside of APOC. The majority (64%) of facilities offered AFS. At facilities offering AFS, most (74%) did not offer services daily, instead offering them only certain days or times of the week. Most facilities reported providing adolescent SRHS, frequently including STI screening and treatment, SRH education, and condoms. The majority of facilities reported providing condoms (65%), FP (60%), and STI screening and treatment (67%) daily, but some clinics reported they never offer condoms (11%) or FP (14%) to adolescents. SRH services offered did not differ by clinic size.

**Table 1:** SRH facility survey data (*n* = 102).

**Survey questions**	**n (%)**
Facility received APOC training for HCWs?	
Yes	57 (56)
Facility received additional SRHS training outside APOC?	
Yes	11 (11)
Are AFS Available?	
Yes	65 (64)
How often are AFS available?	
Every day	17 (26)
On select days	48 (74)
Which adolescent SRHS are provided at this clinic?	
SRH education	98 (96)
Family planning	85 (83)
Condoms	93 (91)
STI screening and treatment	100 (98)
Pregnancy tests	90 (88)
Partner HTS	87 (85)
PMTCT referral	83 (81)
Caregiver required to be present when providing SRHS to non-majority (non-emancipated) adolescents?	
Yes	22 (22)
No/it depends	80 (78)
How often are condoms provided to adolescents?	
Every day	66 (65)
Once every week	2 (2)
Once every 2–4 weeks	1 (1)
Once every month	17 (17)
Once every 3 months	5 (5)
Never	11 (11)
How often is family planning provided to adolescents?	
Every day	61 (60)
Once every week	1 (1)
Once every 2–4 weeks	3 (3)
Once every month	17 (17)
Once every 3 months	6 (6)
Never	14 (14)
How often is STI screening treatment provided to adolescents?	
Every day	68 (67)
Once every week	5 (5)
Once every 2–4 weeks	2 (2)
Once every month	20 (20)
Once every 3 months	7 (7)
Never	0 (0)

### Qualitative Results

Among the 40 ALHIV (10 per facility) who participated in IDIs (Table 2), the median age was 16 years (IQR: 15–17) and the majority were female (68%). All but four were currently enrolled in school. The majority (62.5%) of ALHIV were single at the time of the IDI. Most (70%) had parents or guardians who were also living with HIV. Among the 40 caregivers (10 per facility) who participated in IDIs, the majority were biologic parents (60%) and female (80%). The median age of caregivers was 46 years (IQR: 37–54) and fewer than half (40%) were employed. Nearly all (95%) were caring for an adolescent who was aware of their HIV status. Caregivers who were living with HIV had been taking antiretroviral medications (ARVs) for a median of 6 years (IQR: 4–10 years). There were 16 caregiver/ALHIV dyads (40%) who participated in the study. In addition, 39 HCWs (counselors (39%), nurses (18%), clinical officers (15%), lab technicians (10%), and other HCWs (18%) participated in 4 FGDs (1 per facility with up to 10 participants in each FGD). HCWs had a median of 3 years (IQR: 1–6 years) of experience providing care to ALHIV. Almost half (49%) of HCWs had received training on AFS but few (13%) had received training on SRHS provision.

**Table 2:** Sociodemographic characteristics of FGD and IDI participants.

	**Population**		
	**Adolescents**,	**Caregivers**,	**HCWs**,
	***N* = 40**	***N* = 40**	***N* = 39**
Characteristic	Median (IQR) or n (%)		
Female	27 (67.5)	33 (82.5)	29 (74.4)
Age (years)	16 (15–17)	45.5 (37–53.5)	33 (28–42)
Education^*^			
Primary	20 (50.0)	20 (50.0)	2 (5.1)
Secondary	15 (37.5)	13 (32.5)	8 (20.5)
College/polytechnic	1 (2.5)	4 (10.0)	29 (74.4)
Not currently enrolled	4 (10.0)	–	–
None	–	3 (7.5)	–
Parent/guardian with HIV	28 (70)		
Receiving HIV care	27 (96.4)	–	–
Relationship status			
Single	25 (62.5)	–	–
Steady boyfriend/girlfriend	14 (35.0)	–	–
Married	1 (2.5)	–	–
Employed	–	16 (40.0)	–
Relationship to adolescent			
Parent	–	24 (60.0)	–
Aunt/uncle	–	4 (10.0)	–
Sibling	–	3 (7.5)	–
Grandparent	–	5 (12.5)	–
Other relative	–	4 (10.0)	–
Characteristics of caregiver's ALHIV^**^			
Female	–	25 (62.5)	–
Age	–	16 (14.5–16)	–
Education^*^			
Primary	–	26 (65.0)	–
Secondary	–	12 (30.0)	–
College/polytechnic	–	1 (2.5)	–
Other	–	1 (2.5)	–
Aware of HIV status	–	38 (95.0)	–
Taking ARVs	–	40 (100.0)	–
Years taking ARVs	–	6 (3.5–10)	–
HCW cadre			
Nurse	–	–	7 (17.9)
Counselor	–	–	15 (38.5)
Clinical officer	–	–	6 (15.4)
Lab technician	–	–	4 (10.3)
Other^***^	–	–	7 (17.9)
Years employed at current clinic	–	–	2 (2–5)
Years providing HIV care	–	–	4 (2–9)
Years working with adolescents	–	–	3 (1–6)
AFS training	–	–	19 (48.7)
SRH training	–	–	5 (12.8)

* *Education: For adolescents, current enrollment; for caregivers and HCWs, highest education attained*.

** *Some caregivers and ALHIV participants were dyads (n = 16)*.

*** *HCWs also included cough monitor (1), data clerk (1), nutritionist (1), peer educator (1), pharmacy technician (2), and triage officer (1)*.

ALHIV, caregivers, and HCWs generally described positive experiences receiving or providing adolescent HIV services at their respective facilities. Adolescents reported they were treated well by HCWs, feeling encouraged, respected, and able to communicate openly with HCWs. In addition to supportive and friendly treatment by HCWs, adolescents stressed their appreciation of adolescent-specific services (although not universally available), such as adolescent clinic days, separate waiting rooms, reduced wait times, and weekend clinic days to accommodate their school schedules. Many caregivers felt adolescent-friendly, supportive services were available to their adolescents. Strong relationships and supportive adolescent HIV service delivery influenced the primary themes that emerged regarding integrated adolescent SRHS within HIV care. When evaluating SRHS access and uptake, three key themes emerged as central to characterizing current experiences: (1) HCWs were the preferred source for SRH information, (2) adolescent autonomy was a facilitator of SRH discussions with HCWs, and (3) ALHIV had variable access to and limited utilization of SRHS within APOC-trained health facilities.

#### Trusted, Non-judgmental Individuals Were Preferred Sources for Receiving SRH Information

Adolescents and caregivers stressed that HCWs should be the primary source of SRH information because they were perceived as the most knowledgeable on the topic, able to provide accurate information, and keep information shared confidential (Table 3).

“*The doctor cannot mislead anyone, they have the right information. They will advise her accordingly.”* (58-year-old aunt).

**Table 3:** Additional representative quotes by theme.

**Theme**	**Participant group**	**Quote**
Preferred sources for SRH information	ALHIV	“*I feel most comfortable talking about it [SRH] here in the clinic because in school, teachers are different and they are never confidential, but you know here I am assured of that confidentiality, so I feel most comfortable talking to the nurse here.”* (16-year-old female)
		“*I have gotten used to coming to this clinic ever since I was diagnosed with the disease, so I can take them anything without fear. I feel very comfortable at this place.”* (17-year-old male)
	Caregivers	“*I feel that it is the health care provider who is most suitable [to talk to ALHIV about SRH]….”* (42-year-old mother)
		“*The health workers already know her status, they can explain to her [about SRH] well.”* (47-year-old mother)
		“*Here [at the clinic] they will get all the services they require….the confidentiality of these teenagers and my adolescent will be kept.”* (26-year-old aunt)
	HCWs	“*I am very comfortable because when we are talking to them about sexual and reproductive health, we are providing them with information on how they can prevent themselves from re-infection. In case there is a problem with sexually transmitted infections, the screening is available, and also the treatment. So that is the information they are given.”* (28-year-old male adherence counselor)
Greater adolescent autonomy was a facilitator of SRH discussions with HCWs	ALHIV	“*The way I am living, maybe having problems with my boyfriend, you know such things I cannot involve my mum.”* (18-year-old female)
	Caregivers	“*There could be some questions that the provider may need to ask the adolescent, maybe if he already has a girlfriend, like if he has started experiencing wet dreams…things of that sort. He may feel shy to talk about them if I am with him in the same room.”* (46-year-old mother)
		“*You know I may not be able to know how many girlfriends he already has but when they are just the two of them, then he may be able to disclose to them if he already has one.”* (46-year-old mother)
	HCWs	“*About sex…they may not want to talk about it when the guardian is there, so I might need to excuse them.”* (42-year-old female nursing officer)
		“*For example maybe when an adolescent wants to go for family planning, the adolescent should be able to decide without the caregiver.”* (44-year-old male data clerk)
ALHIV had variable access to and limited use of SRHS within APOC-trained health facilities	ALHIV	“*They do teach us how to use condoms in order to prevent us from transmitting the disease to other people and they also tell us that if we have sex without condoms then we may suffer from reinfection.”* (17-year-old male)
		“*Now me, as an individual, because I currently have a boyfriend, I am always told that we must use protection so as to prevent pregnancy and other STIs, so through condoms I get to protect myself from STIs and also pregnancy.”* (19-year-old female)
		“*We have not come for family planning, but for partner testing we have been planning to come, but we haven't. I was advised by the clinicians that it is very important….”* (19-year-old male)
	Caregivers	“*Maybe they don't tell us, but I don't think they offer it that much because you know they don't have that much time; they are usually there for such a short time it is not even enough for them to be taken through all that.”* (46-year-old mother)
		“*They usually offer family planning to the adolescents depending on the methods that the girls have chosen or sometimes they involve us as caregivers if we would like for our children to be put on family planning methods.”* (43-year-old female relative)
		“*Just the other day she brought for me condoms that she had been given. She also told me that they were instructed that in case they want to have sex then they were supposed to use condoms to prevent pregnancy.”* (69-year-old father)
	HCWs	“*We said the ABC safer sex method, if [you] cannot abstain you be faithful, and if you cannot be faithful use condom, if you cannot use condom you can disclose, because disclosure is very, very important.”* (56-year-old male nurse)
		“*We tell that being sexually active is okay, but they have to be careful. We tell them that now they are HIV positive, there is re-infection, but there is also STIs, so condom use is a key, and also the partners to be brought for testing.”* (42-year-old female nursing officer)

Caregiver decisions on where adolescents should receive information were focused on the importance of having adolescents receive accurate information, while adolescent decisions about where they get information focused more on comfort and confidentiality. Adolescents were often concerned about who would be a supportive and non-judgmental listener that they could openly ask questions of without feeling embarrassed or shy. Adolescents without strong relationships with HCWs identified others, such as siblings, peers, romantic partners, or caregivers they would seek SRH information from and speak freely with.

“*I only have a sister. I am really free with my sister so with her we can square out our issues, we can talk [about] anything, but I don't like taking with the adults, even mum. When she tells me, I blush so she has a limit... [my brother and sister-in-law] do not know more about [SRH] and also they would think that I am already sexually active.”* (17-year-old female).

While adolescents reported they would feel most comfortable receiving SRH information from HCWs, HCWs highlighted that adolescents can sometimes be hesitant to discuss the topic with them.

“*As a care provider, I am very comfortable [discussing SRH] but the adolescents are the ones who shy off and you find that when it comes to sexual and reproductive health... you keep counseling them until they get used to talking about it.”* (25-year-old female clinical officer).

A few adolescents identified peers or romantic partners as important sources of SRH information because they felt they could discuss the topic openly with them. However, caregivers identified these groups as the least desirable source of SRH information for adolescents, given the high risk of misinformation from peers. Some adolescents also identified caregivers as trusted sources of SRH information, depending on their relationship.

“*I feel free talking to my mother because I spend most of my free time with her and my mother is able to sense fast if something is bothering me, hence I will just feel comfortable discussing with her so that she can make me feel at ease.”* (16-year-old female).

Caregivers were polarized in their desire and comfort providing SRH information to their adolescents. Some caregivers saw themselves as an important information source for their adolescent and emphasized their role in reinforcing the SRH information provided by HCWs.

“*Once the provider has given her [SRH] information [and] she comes home, then I will be able to build on that... I will also tell her that I am just a girl like her so we can just talk.”* (50-year-old mother).

Other caregivers, especially those of the opposite sex of their adolescent (*i.e.*, male caregivers of adolescent females), were less likely to feel comfortable discussing SRH with their adolescents.

“*...there are some issues that are a bit hard for a man to talk about with his child, especially those that involve girl[s]...those are best left for women who can easily handle them.”* (69-year-old father).

While most HCWs reported feeling comfortable discussing SRHS with older adolescents, some were less comfortable discussing SRHS with younger adolescents, initially limiting the information discussed with them and only later realizing these adolescents were also in need of comprehensive SRHS.

“*You get a 10-year-old girl, you want to educate her on condom use and family planning and the next question or what she is going to tell you is I don't have a sexual partner, why should I know all these?...then at times, you find that you thought someone was naïve and then the next time she surprises you, she is expecting.”* (26-year-old female clinical officer).

#### Greater Adolescent Autonomy Was a Facilitator of SRH Discussions With HCWs

Many caregivers attended clinic visits with their adolescents and were particularly engaged with HIV treatment challenges and progress.

“*I must have some say over his medical care. For example, if he falls sick I have to come to the clinic to supervise his treatment. I must also come the day that he is coming to know about his CD4 results because I want to know how he is progressing with his treatment.”* (77-year-old grandmother).

While many caregivers were particularly active in their adolescent's HIV care, they did not identify the same need for engagement in all their health services. Adolescents, caregivers, and HCWs identified conversations about SRHS as important instances where adolescents should be more autonomous in their health care. Each group felt adolescents should meet privately with HCWs to discuss these topics, in order to facilitate open communication, which they thought might be hampered with a caregiver present.

“*You find that...there is a part where you ask if they are sexually active, and the child doesn't answer if the parent is there. But if it's just the healthcare provider, they will be very free.”* (32-year-old female adherence counselor).

The majority of caregivers cited SRH discussions between adolescents and HCWs as the most common reason they are asked to leave the exam room.

“*I was told to wait outside as she talked with the doctor... [they were talking about] her sexual behaviors. I had to leave for her to be free with the doctors. At her age if she is sexually active, she wouldn't share such information in [my] presence. That is between her and the doctors.”* (58-year-old aunt).

While often participants cited adolescent shyness as the primary motivator for autonomous visits with HCWs to discuss SRH, one caregiver emphasized their own discomfort as the main reason they would prefer adolescents and caregivers both discuss adolescent SRH with the HCW alone.

“*Sometimes the [HCW] might want to ask me her progress or how her sexual health is. You know if he asks me in the presence of the child then I might also feel shy talking about it.”* (42-year-old mother).

#### ALHIV Had Variable Access to and Limited Use of SRHS Within APOC-Trained Health Facilities

While all adolescents had received some information and education about SRH, their access to additional services and the types of information they had received varied greatly. Most adolescents described exclusively receiving information about abstinence first, and to use condoms only if abstinence was not possible. Only one adolescent mentioned receiving information about pre-exposure prophylaxis (PrEP). Information on condom use often incorporated messages not just about transmission to others, but also around prevention of reinfection.

“*They tell us that we should take care of ourselves, we should not have sex like that, we can use condoms to prevent reinfection and so we do not infect others.”* (15-year-old female).

Caregivers similarly reported that SRH education often revolved around abstinence and the use of condoms to prevent reinfection, HIV transmission, or pregnancy. While this information was widely available, information about STIs, FP options (other than condoms), and STI, pregnancy and partner testing was provided inconsistently. Few adolescents reported they had received information about all of these items, most reported learning about a few or none at all.

“*What they talk about is the abstinence of sex. You can abstain or you can use the condoms...but they don't talk about family planning...they don't urge us.”* (18-year-old female).

Few adolescents reported actually utilizing SRHS other than asking for information. While many reported they would be comfortable accessing such services, they were not currently doing so. Many caregivers did not know about the SRHS their adolescents were using. One caregiver noted that her adolescent did have access to SRHS but did not need these services yet.

“*She also told me that family planning options are also available that one can use if interested, but she told me that she has no interest when it comes to that and I also told her that there is a time for everything. It will reach a time that she will now need to use the contraceptives.”* (42-year-old mother).

The only SRHS adolescents reported using included condoms, oral contraceptives, pregnancy testing, and sex education. No one reported using STI or partner testing services, despite HCWs reporting that they offer these services to adolescents.

Some HCWs identified the SRH component of the checklist in the APOC as useful for guiding their approach to providing adolescent SRHS by improving their explanations of adolescent development and SRHS to adolescents. Other HCWs stated that APOC helped them make adolescents feel more comfortable receiving SRHS. While the APOC helps guide HCWs to provide adolescents with more SRH information, some HCWs were concerned this information was not translating to improved adolescent decision-making about SRH.

“*...the information we are supposed to be giving to adolescents so they can make an informed decision is being given. But them taking it and practicing it, we are not seeing.”* (42-year-old female nursing officer).

Another HCW reported challenges utilizing the APOC SRH checklist components for young adolescents who did not yet have sexual partners. Some HCWs felt this information was not relevant to younger adolescents and might actually encourage them to initiate sexual activity before they are ready.

“*...sometimes I feel like I am putting this [SRH] information or opening their minds to what they are not prepared for, then it is like they are seeing that they need to be doing these things. They even ask themselves why they are late because it is like they are expected to have started.”* (26-year-old female clinical officer).

## Discussion

This analysis highlights the importance of consistent access to adolescent-friendly SRHS for successful integration and uptake of SRHS within HIV service delivery systems and identifies existing implementation gaps. Facility surveys indicated STI screening and treatment, SRH education, and condoms are generally available to adolescents, but some facilities still report never offering contraceptives (including condoms). Similarly, our qualitative findings supported that SRH education about abstinence and condoms was widely available but other services such as FP methods, pregnancy testing, and Undetectable=Untransmittable (U=U) or PrEP for HIV prevention were inconsistently reported as available to ALHIV. The SRH portion of the APOC checklist was generally helpful for HCWs to feel more comfortable providing these services.

Adolescents and caregivers identified HCWs as the most suitable individuals to provide adolescent SRHS. While caregivers emphasized providers' expertise and confidential services, adolescents stressed the importance of feeling comfortable to talk openly with HCWs as a key factor in accessing SRHS. Strong relationships between ALHIV and others were a key factor in determining their comfort level discussing SRH with a specific individual or group. Adolescent autonomy was important for facilitating candid discussions of SRHS between adolescents and HCWs.

For ALHIV, having a positive and open relationship with HCWs was an important influencer of their comfort discussing SRH. Previous studies have similarly found that ALHIV identify amicable providers as a key component of adolescent-friendly SRHS (21, 22). This study also found that non-judgmental HCWs were critical to foster relationships that would allow adolescents to ask questions about SRH. HCW discomfort or personal beliefs and values about adolescent SRH needs and rights have been found to be a barrier to adolescent SRHS provision in other settings (19, 23). In contrast, HCWs in this study believed it was their responsibility to share SRH information and provide these services regardless of their beliefs. This finding was surprising and possibly attributable to the APOC training provided regarding adolescent SRHS provision.

Adolescent autonomy emerged as an important theme in improving access to SRHS. During adolescence, major changes in caregiver-child relationships and communication occur as adolescents seek greater autonomy and independence in decision-making (24, 25). Our finding of autonomy as a facilitator of SRHS discussions between HCWs and adolescents reflects adolescents' desire for independence, particularly for topics such as sexuality and relationships. To improve access and uptake of SRHS for ALHIV, it is important that adolescents are supported by both HCWs and caregivers in ways that respect and foster their growing autonomy. This should include allowing adolescents to meet independently with HCWs to discuss SRHS. These findings can help inform the delivery of adolescent SRHS to maximize uptake in Kenya and in other SSA settings where SRHS are being integrated within HIV care.

Prior evaluations have reported SRHS uptake can be improved when providers are trained in AFS, when outreach activities engage adolescents to inform them of available services and encourage utilization, and when community members support the provision of adolescent health services (26–28). While the APOC promotes AFS and the integration of HIV and SRHS delivery, further improved SRHS might occur as the APOC has continued to be implemented and reinforced at facilities throughout Kenya after this study's completion. The strong, cross-cutting themes identified in this study provide key recommendations for improving APOC delivery. To actualize the SRH rights of adolescents, adolescents should consistently be provided accurate SRH information and access to SRHS. This will require expanded political and social support and increased external and domestic funding to improve adolescent SRHS and APOC delivery (29). Additionally, adolescent-friendly SRHS trainings must be provided regularly to HCWs in-service that encourage them to examine their attitudes and beliefs toward adolescent sexuality and SRHS (30, 31). To further improve adolescent SRHS uptake and positively influence adolescents' sexual behavior, caregivers should be encouraged to support HCW messaging to adolescents about SRHS to foster communication about SRH (32, 33).

This study had a number of limitations. The facility surveys were conducted at larger facilities and SRHS offered might be different at smaller facilities. Adolescents who participated in the study were actively involved in HIV care and caregivers were engaged in their adolescent's HIV care and willing to come to the facility for an IDI. Therefore, study results may not be representative of perspectives of ALHIV not currently engaged in care and caregivers not frequently attending the clinic with their adolescents. Additionally, all IDIs and FGDs were conducted in one county in Kenya among APOC facilities and might not be generalizable to other settings. However, these facilities were selected from a region in Kenya with a high HIV burden and it is expected that the perspectives shared by participants would be similar to other ALHIV, caregivers, and HCWs in these regions. Adolescent SRHS offered at non-APOC facilities might be more limited and should be explored further. Social desirability bias is a concern, given participants largely highlighted only positive attributes of the facilities and comfort providing or receiving adolescent SRHS. To mitigate this, the topic guides probed what participants did not like about receiving HIV care and SRHS services at their facilities and what could be improved.

## Conclusion

Our results indicate the importance of utilizing HCWs in provision of adolescent-friendly SRHS services as well as ensuring consistent and comprehensive integration of SRHS provision into HIV treatment and care. While some SRHS were reported frequently available to adolescents, they were not consistently available across all facilities. Furthermore, while ALHIV, caregivers, and HCWs noted feeling satisfied with the APOC and currently available SRHS provision, qualitative evidence did not support that substantive SRHS were being provided to or used by ALHIV. To better meet the needs of ALHIV, consistent and comprehensive SRHS should be provided in the future while continuing to build upon the existing foundation of trust between HCWs, ALHIV, and caregivers.

## Data Availability Statement

The raw data supporting the conclusions of this article will be made available by the authors, without undue reservation.

## Ethics Statement

The studies involving human participants were reviewed and approved by The University of Washington Institutional Review Board (IRB) and the Kenya Medical and Research Institute Ethical Review Committee (ERC). Written informed consent to participate in this study was provided by the participants' legal guardian/next of kin.

## Author Contributions

GJ-S, PK, KB-S, and BS served as the principal investigators of the study, providing leadership in study design, implementation, and data analyses. BS, JK, CM, and JI coordinated the field team in Kenya with technical assistance from IM and T-HC. SL, HM, KW, KB-S, and JN analyzed the data. SL wrote the first draft of the manuscript. JN, PK, AK, GJ-S, and KB-S reviewed the manuscript and provided scientific oversight. All authors contributed to and approved the final manuscript.

## Conflict of Interest

The authors declare that the research was conducted in the absence of any commercial or financial relationships that could be construed as a potential conflict of interest.
